# Mode selection of post-earthquake recovery and reconstruction of traditional villages using dependency analytic process: taking Xiluo-Buzi village in the 2022 M6.8 Luding earthquake as an example

**DOI:** 10.3389/fpubh.2023.1240573

**Published:** 2023-08-31

**Authors:** Chao Huang, Jian Qiu, Tianmin Huang

**Affiliations:** ^1^School of Architecture, Southwest Jiaotong University, Chengdu, China; ^2^School of Mathematics, Southwest Jiaotong University, Chengdu, China

**Keywords:** seismic hazard, damage characteristics, traditional village, cultural heritage protection, post-earthquake recovery and reconstruction, influencing factors, prediction model

## Abstract

**Introduction:**

Traditional villages are precious historical and cultural heritage sites. The selection of post-earthquake recovery and reconstruction (PERR) mode directly affects the village cultural heritage protection and the development direction of post-disaster reconstruction. A scientific and comprehensive feasibility evaluation for selecting the PERR mode of traditional villages can provide sufficient evidence for the recovery efforts in earthquake-stricken villages.

**Method:**

The author summarizes three PERR modes and constructs an evaluation index system for the selection of PERR modes of traditional villages. Based on the interrelationship of the indicators, the author has preliminarily established the Dependency Analytic Process (DAP), Based on this method, a model of traditional village PERR mode selection is constructed, and an empirical analysis is carried out in the case of the earthquake-stricken area of Xieluo-buzi Village in 2022 M6.8 Luding earthquake, to discuss the selection of PERR modes of traditional villages.

**Results:**

The authors have explored the application of the DAP in the selection of PERR modes for traditional villages and verified the effectiveness of the method. Since a large amount of actual research work is required to conduct an assessment, it is believed that with the widespread applications of the DAP, its superiority and practicality will be further demonstrated.

**Conclusion:**

The protection of traditional villages is a dynamic protection process, in which the will of the indigenous people is respected, the social network of the indigenous people is maintained, and the fair rights of the indigenous people to participate in the implementation of the project and to enjoy the preferential policies and resource benefits are guaranteed, as they are the real main body of the heritage protection, so that the traditional village ethnic heritage can be inherited and developed permanently in the protection. DAP is applicable to the comprehensive evaluation of multiple factors, particularly in situations where the importance of the indicators is difficult to be distinguished from each other. This is a new method to determine the weight vector, which has a broad application prospect.

## 1. Introduction

Destructive natural disasters considerably affect the world (1, 2). In 2023, two major earthquakes occurred worldwide: the 7.8 dual-type earthquake in Turkey and the 7.2 magnitude earthquake in Tajikistan. Sudden major earthquakes cause not only a certain number of casualties but also extensive damage to the physical environment and chaos and stagnation in the social order. Post-earthquake recovery and reconstruction (PERR) includes environmental remediation and ecological construction. It also involves the future development direction concerning the social structure and daily lives of the people in the disaster area, which are widely valued in affected regions.

After the Wenchuan earthquake in 2008, many experts and academics studied the reconstruction mode of rural housing (3, 4). However, in reality, most traditional villages are treated as ordinary rural houses, and some cultural symbols are added during the reconstruction process, transforming genuine heritage into inauthentic cultural artifacts. Thus, the inherent heritage value of the traditional village itself is lost. Traditional villages are precious historical and cultural heritage sites. In the PERR, these sites are supported by the key technology of heritage protection and planning for historical environment recovery and reconstruction. Traditional villages are important carriers of the protection, inheritance, and economic development of ethnic culture (5–7). Their ethnic significance creates the uniqueness and sensitivity of traditional village cultural heritage (8). The trust crisis leads to difficulties and unstable factors in the recovery efforts for earthquake-stricken people (9). However, the PERR of traditional villages can affect not only the quality of life of indigenous people in the future, but also affects the protection of the villages’ material and intangible cultural heritage and the historical environment. Selecting the reconstruction mode can directly affect the development direction of PERR.

Domestic and international experts and scholars have made progress in the research on PERR evaluation. Yang Yueqiao and others constructed a comprehensive evaluation framework for PERR (10); Zabihullah Sadiqi developed a framework for community participation with five common barriers and proposed it as a pragmatic solution to delivering sustainable post-disaster reconstruction projects (11); Zhao Liang combined the domestic and international analysis of the implementation evaluation of post-disaster reconstruction planning. They emphasized the importance of cultural preservation in the evaluation (12); Yang Yueqiao and others discussed “People first” as one of the basic principles of post-disaster reconstruction from the satisfaction of earthquake victims (13). Literature surveys have found that most post-disaster reconstruction focused on evaluating the results of post-disaster reconstruction to emphasize the problems and effectiveness of strategies, and there is little research have been conducted on the selection of post-disaster reconstruction modes, especially for traditional villages with special heritage values. However, choosing an appropriate post-disaster recovery and reconstruction mode based on the heritage value is also a point of misunderstanding and omission in treating traditional houses as ordinary rural houses reconstruction.

Therefore, a scientific and comprehensive feasibility evaluation for selecting the PERR mode of traditional villages can provide sufficient evidence for the recovery efforts in earthquake-stricken villages. The author researches and lists the influencing factors of post-disaster reconstruction mode selection, deeply analyzes the influence of each influencing factor on post-disaster reconstruction decision-making, and explores the selection of PERR modes for traditional villages by constructing an indicator system and determining the weights by using the Dependency Analytic Process (DAP) established by the author.

## 2. Evaluation for selecting PERR mode for traditional village

### 2.1. PERR mode for traditional village

The post-disaster reconstruction modes in various parts of the world mainly include on-site reconstruction, off-site reconstruction, partial on-site, and partial off-site reconstruction (14–16). Regard to the PERR modes for traditional villages, the authors have summarized the relevant research results (Table 1).

**Table 1:** Overview of existing research on PERR modes for traditional villages.

Author	PERR mode	Characteristic	Key point
Qiu (17)	On-site recovery	The form of the village itself after the earthquake is relatively intact and has high preservation value, and the environment and spatial pattern of the base have not changed significantly.	Local materials and traditional techniques are used, applied to avoid secondary damage, and a scientific and rigorous attitude is adopted in the restoration.
	On-site expansion	Transfer the center of village life through expansion and reconstruction to protect the old village. Integrate cultural tourism in old villages with accommodation, entertainment, and recreation in new villages.	Bringing opportunities for tourism development but may create conflicts between conservation and development. Clarify that the villagers are the main body of construction and operation and coordinate the interests of all parties.
	On-site reconstruction	The village is severely damaged but the original base is usable, After the residents are relocated to the new site, the original site will be rebuilt as a tourist attraction for earthquake sites and ethnic tourism.	Usually, the purpose is to develop ethnic culture tourism, but the rebuilt ethnic characteristic space is easy to be commercialized and cannot become a true carrier of ethnic culture without indigenous people.
	Off-site reconstruction	The village is completely destroyed or the base where it is located is destroyed. The overall indigenous people are relocated.	How to reflect and pass the traditional culture on is the difficulty of this type of reconstruction.
Shi (18)	On-site reconstruction	On the basis of preserving the national characteristics, the on-site reconstruction introduces a modern lifestyle, which improves the living comfort of residents in the affected areas.	Scientific reconstruction should adhere to authenticity and integrity, which may result in a certain degree of damage to the original neighborhood relationships.
	Off-site construction	On the basis of the fact that the original site is severely affected by the disaster and is located in the fracture zone area.	It requires support and efforts in terms of policy and funding. The original historical sites are gradually disappearing.
Long (19)	On-site reconstruction	The ecological environment and basic layout of the village have not changed significantly, and the dwellings have the conditions for reconstruction, which can be reconstructed using the original materials.	The culture and ecology are well preserved. However, disaster prevention, transportation, and other aspects are relatively primitive, and there are hidden dangers.
	New relocation	The village buildings have been seriously damaged or collapsed, and the village needs to be completely or mostly rebuilt.	The reconstruction is more modernized. The negative effect of village cultural reconstruction outweighs the positive effect.
	Off-site reconstruction and on-site renovation	The village buildings are seriously damaged, and reconstruction is carried out on a nearby site, preserving the old village as a cultural heritage site.	After reconstruction, it will be harmonized with the style of the landscape and environment of the original village.
Liu (20)	Off-site construction	It includes unified planning and self-construction, unified planning and construction, renewed the living and public spaces.	It should understand the needs of the victims, fully mobilize their enthusiasm and avoid conflicts.
	On-site reconstruction and reinforcement	It greatly preserves the local village dwellings, texture, neighborhood relationships, and traditional culture.	This is the most important mode of post-disaster reconstruction, which minimizes the cost of reconstruction.
	Self-organized renewal	Government-led to resident-led, actively guiding victims to rebuild their homes by self-organized renewal.	It gives full consideration to the wishes of residents and reduces the material and spiritual losses caused by the disaster.

From the research of these scholars, it can be seen that PERR of traditional villages is the protection and redevelopment of traditional cultural heritage, and the selection of a suitable reconstruction mode will have a strong guidance for the subsequent development of the villages. Respecting the research results of these scholars as the premise, the authors believe that there are four principles to be followed of the PERR of traditional villages. Firstly, it is important to pay attention to scientific site selection, which needs to be considered for both on-site and off-site reconstruction. Secondly, scientific recovery should be carried out to protect the integrity and originality of the cultural heritage in order to perpetuate the culture. Third, the infrastructure functions, such as production and living should be perfected, and the quality of life should be upgraded. Finally, according to the local ethnic characteristics, combined with the post-earthquake resources, the positioning of the development of the traditional village should be clearly defined, and the overall planning should be carried out to create traditional cultural characteristic industries and protect them during development.

According to the above principles, combined with the previous laws of the PERR and the requirements for scientifically restoring the heritage of traditional villages, and based on the research of other scholars, the authors have implemented the three modes of on-site reconstruction, off-site reconstruction, and partial on-site and partial off-site reconstruction in practice, namely: (1) restoring traditional villages at the original site while preserving the original layout as much as possible, (2) relocating traditional villages to a new location while transforming the original site into a village tourism area, (3) organically evacuating the core of the village while considering the wishes of indigenous residents from multiple perspectives.

#### 2.1.1. Mode 1: restoring traditional villages at the original site while preserving the original layout as much as possible

With the aim of preserving cultural heritage, PERR should strive to maintain the original form, original materials and original craftsmanship technology of traditional villages. Altering the traditional village’s form, spatial layout, and landscape characteristics must be avoided. These alterations can cause secondary damage to the natural environment (17). The traditional buildings in a village are the result of traditional construction techniques. The restoration and preservation of heritage sites require more complex repair and reinforcement techniques than those of ordinary rural house reconstruction. Thus, the former entails greater financial investment than the latter.

Restoring the original site can greatly restore the cultural heritage value of traditional villages. However, the process may face difficulties in terms of technical expertise because of the lack of skilled personnel and insufficient funding.

#### 2.1.2. Mode 2: relocating traditional villages to a new location while transforming the original site into a village tourism area

In this mode, the indigenous residents are relocated to a new location, while the original site is transformed into a village tourism area by a platform company. This mode aims to create a traditional village leisure tourism area with ethnic characteristics, integrating culture and tourism while protecting the overall historical landscape of the original village. This mode guides industrial transformation and alleviates the significant financial pressure of recovering the old village due to funding constraints.

The overall relocation method maintains the integrity of relationships of family, friends, and neighborhood within the village, preserves the non-material cultural heritage, and optimizes the living environment. However, the newly built villages inevitably lack the original form and original materials left by traditional houses. The integrity of the historical landscape, which encompasses the livability of traditional villages, is completely lost, resulting in a decline in heritage value.

#### 2.1.3. Mode 3: organically evacuating the core of the village while considering the wishes of indigenous residents from multiple perspectives

In PERR, the demands of the affected residents may vary. Therefore, a detailed assessment can be conducted to determine the extent of damage to each household identify the specific needs for PERR, and distinguish and record the number of households that need to be repaired at the original site or relocated to a new area. A grid-based management approach can be implemented to facilitate organic dispersal and partial adjustments, relocating. Thus, the core living area of the village is relocated while improving the infrastructure and optimizing the living environment. This mode aims to maintain the original living conditions of the village.

However, this mode can easily disrupt the original social order, and the existing social form and familiar and trusted relationships can be changed. These changes can result in a loss of production organization and a gradual fading of ethnic customs, traditions, and religious practices. It may fulfill the demands of most affected residents. However, the risk of gradually eroding the traditional village’s material and non-material cultural heritage value exists.

### 2.2. Evaluation indicator system of post-disaster recovery and reconstruction mode selection

#### 2.2.1. Analysis of evaluation indicator system structure

The following contradictions in PERR were found by the experience of post-disaster reconstruction in the past: the contradiction between the disaster victims who longed for a better life and protection of traditional villages, the contradiction between the existing social reconstruction capacity of the village and the high protection costs, and the contradiction between the new resettlement sites and the authenticity of the village. The above contradictions affect the PERR mode selection.

The author believes that each of these contradictions should not be measured independently. However, the interdependence and support between them, which constitutes the principle of the evaluation index system in this article, should be evaluated. Therefore, the author believes that the evaluation index system for selecting PERR modes of traditional villages is determined by the heritage attributes of the village and the indirect influencing factors of post-disaster reconstruction.

##### 2.2.1.1. Heritage value of traditional villages

**Figure 1:**
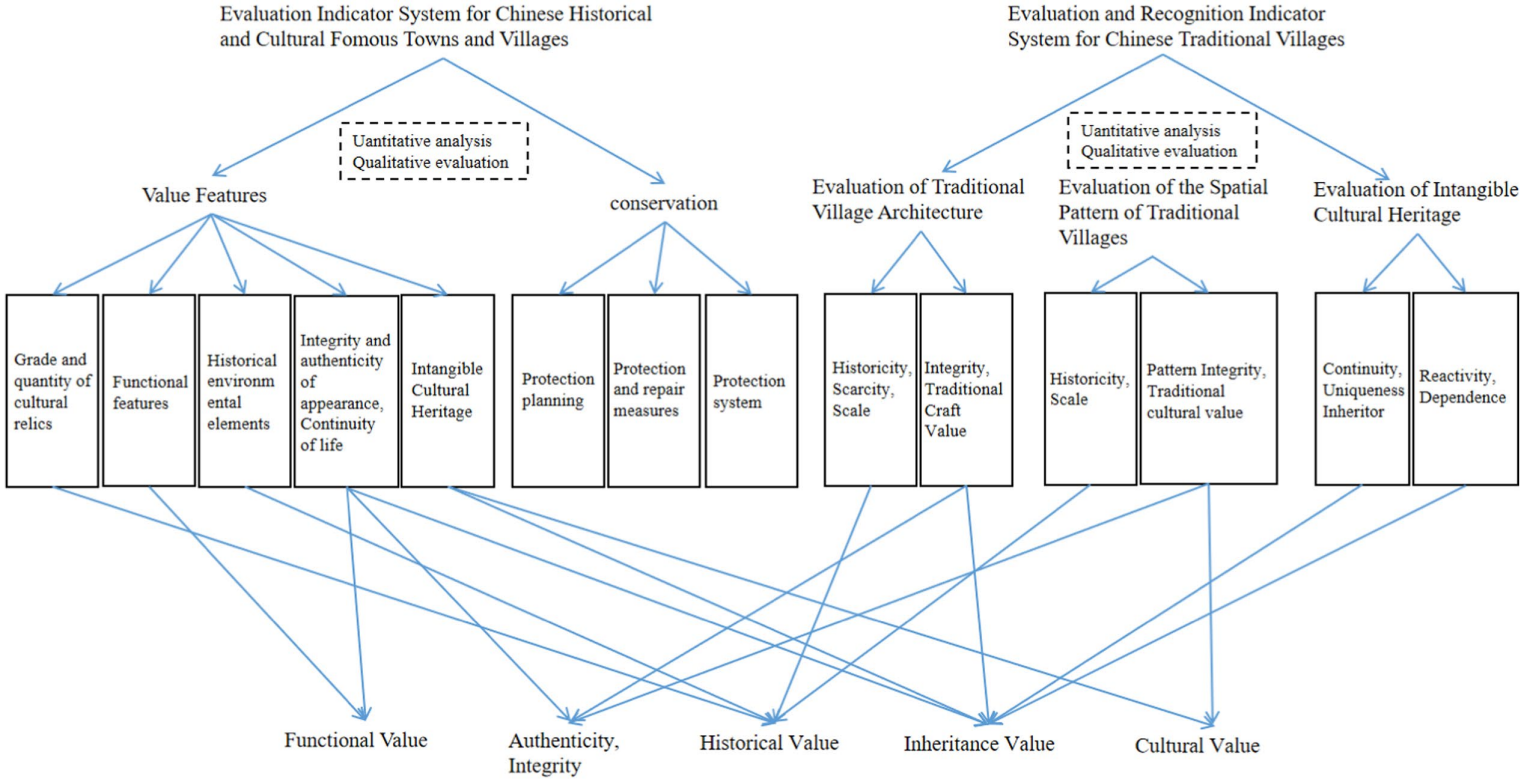
Criteria for evaluating and identifying the value of famous historical cultural towns, villages, and traditional villages in China [Adapted from Zhao, JX. (22)].

Traditional villages have unique heritage attributes, and describing and evaluating their value is a difficult and complex issue for experts and scholars worldwide and even countries (21). Zhao Jingxue used CiteSpace software to sort out relevant domestic and international literature and also divided the evaluation objectives of traditional village values into three aspects: preservation, development and utilization, and hierarchical classification (22); Huang Tingwan systematically sorted out the phased characteristics of the changes in the selection of indicators and value evaluation of Chinese traditional villages (23). Currently, China has formulated the “Evaluation Indicator System for Chinese Historical and Cultural Famous Towns and Villages (Trial)” and the “Evaluation and Recognition Indicator System for Chinese Traditional Villages (Trial)” (Figure 1). In PERR, the heritage value of traditional villages is an important factor to consider when choosing the reconstruction modes. On-site restoration is mostly the first choice for villages with high levels of protection and cultural heritage value. The possibility of off-site reconstruction is often considered for small villages with low levels of heritage protection value and completely unsuitable environments for living, the possibility of off-site reconstruction is often considered.

##### 2.2.1.2. Indirect factors of recovery and reconstruction

###### 2.2.1.2.1. Post-disaster situation

The assessment of the post-disaster situation determines the plan for PERR, and the extent of damage is an important criterion (24). For villages with relatively minor damages, only a few houses are affected, and public facilities remain basically intact. The relatively high selection is on-site recovery and reconstruction.

###### 2.2.1.2.2. Social disaster relief work

As opposed to regional work, social work has made remarkable achievements in attaining disaster resilience. Human relationships play an important part in PERR. That is, social capital plays an important role in meeting the needs of victims, innovating social management in disaster areas, and promoting the development of disaster areas. Considerable research attention is currently given to whether pre-disaster levels can be quickly and successfully restored under the influence of social capital (25, 26), the specific functions of social capital in PERR (27, 28), and the influence of social capital in PERR in ethnic areas (29). Many existing studies show that the areas with abundant social capital have a stronger ability to resist disaster damage and recover quickly (30). Social capital has considerable effects on trust, public participation, and social behavior norms in the context of disaster impact and recovery (31–33). Therefore, social capital with healthy social relationships is an important force for disaster resilience and development orientation. It plays a considerable role in PERR and is an important factor in the protection and inheritance of ethnic culture and economic development.

###### 2.2.1.2.3. Management and policy support

In the case of the Wenchuan earthquake, China’s PERR efforts demonstrated characteristics such as concentrated resources, efficient emergency response, and relatively short reconstruction time. The miracle of basically completing the reconstruction of disaster areas in just over 3 years was achieved because of efficient management and policy support. In the global trend of advocating a comprehensive “top-down” management for PERR, many experts and scholars have recently carried out policy innovations and practical examinations in PERR domestically and internationally (17, 34). The level of policy support and compensation systems for on-site restoration and relocation also affect the PERR mode selection.

###### 2.2.1.2.4. Physical environment impact

Beichuan Town was left in ruins after the Wenchuan earthquake caused by the Beichuan-Yingxiu active fault zone cutting through the town. The research data from major earthquakes worldwide also show that avoiding active fault zones is usually regarded as an important basis for urban site selection and future planning to avoid disaster risks (16, 17, 35). Thus, PERR mode selection is also influenced by the physical geography and environmental conditions of the area.

###### 2.2.1.2.5. Development prospects and benefits

The rapid development of urbanization has led to the gradual decline of traditional villages and the risk of culture loss. The earthquake disaster has once again dealt a heavy blow to these villages. PERR should focus on the heritage value of traditional villages. However, it is also a development opportunity for enhancing the acceleration of rural revitalization. It should not only restore normalcy but also establish an enhanced new normal. It should also create safe, sustainable, resilient and adaptable rural communities. Therefore, future development benefits after recovery and reconstruction are also a concern for people.

#### 2.2.2. Construction of evaluation indicator system

In summary, the author combines the heritage protection of traditional villages with the indirect impact of the reconstruction based on domestic and international research of evaluation indicators of PERR and on-site surveys and analyses. The author also believes that the evaluation for selecting PERR modes of traditional villages involves factors such as the cultural heritage value of the village, post-disaster situation, social capital, policies and guarantees, physical geography and environmental conditions, and socioeconomic benefits. Therefore, the evaluation indicator system for PERR mode selection is established (Table 2).

**Table 2:** Evaluation indicator system for PERR mode selection of traditional village.

Target layer	First-level	Serial No.	Second-level indicators
Mode Selection of post-disaster recovery and reconstruction of traditional village (G)	The value of Traditional village cultural heritage (A)	A1	Protection level of traditional villages
		A2	Relevance to significant historical events, revolutionary movements, and famous figures
		A3	Importance of ethnic culture
		A4	The uniqueness of ethnic and local characteristics
		A5	Representativeness of traditional village’s regional architectural style and spatial layout
		A6	Uniqueness of local materials and craftsmanship
		A7	Presence of inheritor of ethnic, cultural heritage
	Post-disaster situation of traditional village (B)	B1	Integrity of traditional buildings and historical environment post-disaster
		B2	Authenticity of the village
		B3	Scale of the village
		B4	Integrity of public facilities post-disaster
	Social capital of traditional village (C)	C1	Mutual assistance and altruistic behavior within the village’s interpersonal relationship
		C2	Influence and appeal of grassroots village organizations
		C3	Influence of village elites
		C4	Trust and emotional stability of villagers
		C5	Execution capability of non-governmental organizations (NGOs)
		C6	Support for mobilizing private capital
	Management and policy support in PERR (D)	D1	Social impact of the disaster
		D2	Policy support and guarantee for post-disaster reconstruction and rural revitalization
		D3	Fairness of government policies
		D4	Post-disaster execution of government departments
		D5	Level of support from government reconstruction funds
		D6	Trust of affected people in the government
	Physical geography and environmental condition (E)	E1	Permissibility of geological hazard environment
		E2	Accessibility of transportation
		E3	Availability of land resources for reconstruction needs
		E4	Ecological value of the village environment
	Development prospects and benefits (F)	F1	Significant improvement in villagers’ living standards
		F2	Significant increase in villagers’ happiness index
		F3	Influence on the surrounding areas
		F4	Reflection of heritage conservation value
		F5	Value of village cultural tourism and future development planning potential

#### 2.2.3. Scoring criteria

Traditional village PERR mode selection evaluation is based on the village heritage value and post-disaster status quo, with the purpose of selecting the PERR mode and combining it with the actual situation of PERR. Each factor assigns the value according to the degree of support for the selection of the mode. On the basis of relevant domestic and international research and field surveys, the author strives for an intuitive and accurate assessment by setting six value ranges for the support level and establishing a support-level evaluation table (Table 3), and then sub-scoring are scored according to this standard, providing a judgment scale for the final choice and adaptability of PERR.

**Table 3:** Support level evaluation table.

**Evaluation**	**Support**
0	Not supported at all
2	Hardly supported
4	Low support
6	Moderate support
8	Relatively support
10	Heartily supportive

#### 2.2.4. Comprehensive evaluation

After determining the evaluation system and index scoring standard of the traditional village PERR mode selection, a comprehensive evaluation can be conducted on disaster-stricken traditional villages or famous towns and villages. The author participated in the investigation of the PERR of traditional villages in the *M*6.8 Luding earthquake. Understanding the current situation of the disaster and the protection of traditional village cultural heritage, using a new evaluation method - Dependency Analytic Process (DAP), to try to select the optimal recovery and reconstruction program to provide valuable technical support for the reconstruction work in the disaster area, to provide a direction for the choice of PERR mode, as well as to avoid the possible problems that may be encountered during the process of post-disaster reconstruction.

## 3. Evaluation method: dependency analytic process

Weight vectors are crucial in comprehensive evaluations, optimization decisions, and multi-objective decisions involving multiple factors. Many methods for determining weight vectors exist, which can be categorized into two types. The first type is mostly determined by subjective factors, such as the Expert Grading Method. The other type is determined by certain objectivity, such as the Analytic Hierarchy Process (AHP) (36, 37) and Eigenvector Method (EM) (38). A comprehensive evaluation is gradually and widely applied in the field of heritage conservation. Table 4 summarizes the common comprehensive evaluation methods in the field of traditional village conservation both domestically and internationally, summarizes the main features, scope of application, and advantages of each method, and points out the application scope of these evaluation methods in the field of traditional village conservation in a targeted manner.

**Table 4:** Comparison and summary of common heritage evaluation methods.

Method	Main features	Scope and advantages of use
Fuzzy comprehensive evaluation, FCE	Fuzzy mathematics is applied to the method of comprehensive evaluation, and the evaluation results are obtained by establishing a single factor evaluation matrix and fuzzy operation.	It is suitable for the overall evaluation of objects constrained by multiple fuzzy factors. It has advantages in dealing with some qualitative problems that are difficult to precisely quantify. It is mostly used for evaluating the satisfaction of post-disaster reconstruction of traditional villages.
Principal component analysis, PCA	A method based on multivariate statistical analysis, which simplifies the calculation of problems by converting multiple variables into as few new variables as possible.	It is applicable to situations with multiple and complex variables, and it can be able to simplify the complexity, discard the false and retain the true, and grasp the essence and focus of the problem. It is comprehensive and comparable. It is mostly used for evaluating the comprehensive benefits of traditional village reconstruction.
Analytic hierarchy process, AHP	A multi-objective decision-making method that calculates weights by analyzing the importance of factors.	It is a commonly used method to determine the weights, which combines qualitative judgment with quantitative analysis of decision-makers, and the error is small. It is mostly used for evaluating the heritage value of traditional villages.
The Entropy method, EM	An objective weighting method that determines the weight of an indicator by calculating the information entropy of the indicator.	It is suitable for dealing with information that it not completely certain. It is mostly used for evaluating the heritage value of traditional villages.
Delphi method, DM	Also known as the expert scoring method, it is a subjective evaluation method based on experts’ professional knowledge and scientific research experience.	It is a commonly used qualitative evaluation method, which is relatively simple in operation and mainly copes with the management techniques of complex tasks. It is mostly used for evaluating the heritage value of traditional villages.
Contingent valuation method, CVM	The questionnaire design and survey process are inevitably subjective.	It provides very useful decision support for environmental protection and resource management. It is mostly used for evaluating of economic and social values of traditional villages.

In conducting the research on comprehensive evaluation of PERR of traditional villages, the author synthesized the characteristics of various types of comprehensive evaluation methods and found that the current evaluation methods are relatively concentrated, mostly targeting independent evaluation factors without considering the interrelationships among the evaluation factors. In the present study, the author refers to an eigenvector method based on the dependency matrix. This method is a completely objective approach to determining weight vectors. The method is a completely objective approach to determining weight vector. It determines based on the interdependencies among various indicators with clear causal logical relationships in terms of weight vector magnitudes. This novel method is referred to as Dependency Analytic Process (DAP).

DAP is a multi-objective decision-making method that combines qualitative and quantitative analyses. The main idea of this method is to decompose complex problems into several levels and factors. Given the correlation among these factors, a dependency matrix is established based on the dependency degrees between the two indicators. The weights of the indicators are determined by calculating the eigenvalues of the matrix, providing a basis for optimal solution selection.

### 3.1. Constructing the dependency matrix

There are multiple indicators in the comprehensive evaluation, set as $a_{1},a_{2},\cdots a_{n}$ . Generally, these indicators are not completely independent but have a certain dependency on each other. If 10% of the contribution to $a_{i}$ comes from $a_{j}$ , then define the dependency degree of $a_{i}$ to $a_{j}$ as $s_{ij}=0.1$ . Thus, the matrix obtained as:


(1) $$B=\left(\begin{array}{ccc} s_{11} & \cdots & s_{1n}\\ \vdots & \cdots & \vdots \\ s_{n1} & \cdots & s_{nn} \end{array}\right)$$

usually, $s_{ij}$ is not necessarily equal to $s_{ji}$ . We take the interdependence between $a_{i}$ and $a_{j}$ as: $r_{ij}$


(2) $$r_{ij}=\frac{s_{ij}+s_{ji}}{2}=r_{ji}$$

constructing the Dependency Matrix:


(3) $$A=\left(\begin{array}{c} \begin{array}{cc} r_{11} & r_{12}\\ r_{21} & r_{22} \end{array}\;\begin{array}{cc} \cdots & r_{1n}\\ \cdots & r_{2n} \end{array}\\ \begin{array}{cc} \vdots & \vdots \\ r_{n1} & r_{n2} \end{array}\;\begin{array}{cc} \vdots & \vdots \\ \cdots & r_{nn} \end{array} \end{array}\right)$$

since matrix A fully reflects the interdependencies among the indicators, it is called A as the dependency matrix. A is a real symmetric matrix with a main diagonal of 1 and other elements between (0.1).

The sum of the dependency of an indicator on other indicators should be less than 1, otherwise from the perspective of evaluation, this indicator is redundant and can be eliminated. If the sum of the dependency of an indicator on other indicators exceeds 1, it indicates that the dependencies are unreasonable. Therefore, further requirements can be imposed:


(4) $$\overset{n}{\underset{j=1}{\sum }}r_{ij}-r_{ii}<1,i=1,2,\ldots n$$

Therefore, A is a real symmetric matrix with a main diagonal element of 1 and is strictly diagonally dominant. Thus, A is a positive definite matrix, and all its eigenvalues are greater than zero.

### 3.2. Calculating dependency

(a). Calculate the sum of the interdependency between the indicator $a_{i}$ and other indicators, denoted as $b_{i}$ :


(5) $$b_{i}=\overset{n}{\underset{j=1}{\sum }}r_{ij}-r_{ii},i=1,2,\cdots n$$

the larger $b_{i}$ , the more important the corresponding indicator $a_{i}$ is, and thus the greater the weight of indicator.

(b). Make $b_{i}$ arranged in descending order and denote it as $c_{1}\geq c_{2}\geq \cdots c_{n}$ . In other words, the largest $b_{i}$ is $c_{1}$ , the second largest $b_{i}$ is $c_{2}$ , and so on, and the smallest one is $c_{n}$ .

### 3.3. Determine weight

Assume that the eigenvalues of the dependency matrix *A* are sorted in descending order as:


$$\lambda_{1},\lambda_{2},\cdots \lambda_{n}$$

Order:


(6) βi=λi∑j=1n λj,i=1,2,⋯n

due to $\lambda_{1}\geq \lambda_{2}\geq \cdots \lambda_{n}$ , so $\beta_{1}\geq \beta_{2}\geq \cdots \geq \beta_{n}$ .

Assign the weight of indicator $a_{ki}\;$ corresponding to $c_{i}$ as $\beta_{i},i=1,2,\cdots n$ .

### 3.4. Calculate the evaluation result

Calculate the total evaluation value:


(7) $$Y=\beta_{1}a_{k1}+\beta_{2}a_{k2}+\cdots +\beta_{n}a_{kn}+\varepsilon$$

Y is the evaluation result, $\beta_{1},\beta_{2},\cdots \beta_{n}$ is the weight vector calculated from the second step above, $a_{k1},a_{k2},\cdots a_{kn}$ is the reordering of $a_{1},a_{2},\cdots a_{n}$ , based on its dependencies on other indicators and also the scores of each indicator. ε is the error term used to account for the occurrence of special circumstances. For example, after the “5.12” Wenchuan Earthquake, the government wanted to relocate all residents around the Dujiangyan Scenic Area and implement a new urban plan, but the local residents would rather not have any support from the government and resolutely refused the relocation, making it impossible to carry out the relocation plan.

**Figure 2:**
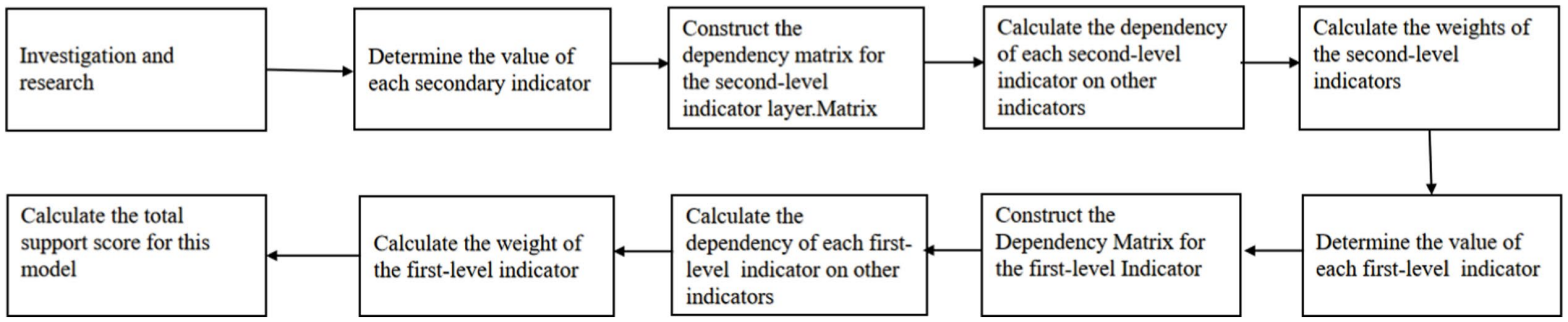
Flowchart of the DAP (Drawn by the author).

This method utilizes the dependency matrix to reflect the importance of indicators, i.e., the magnitude of the weights. The greater the dependency on other indicators, the greater the corresponding weight, and the smaller the dependency, the smaller the weight. The weight vector is determined by relying on the eigenvalues of the dependency matrix. The score in the second-level indicator represents the level of support for the selected mode, calculating the support for each factor in the first-level indicators. The same method as above, determining the weight vector for the first-level indicators, obtaining the final evaluation result (Figure 2).

### 3.5. Application

The DAP is created by the author in the comprehensive evaluation of PERR for traditional villages. In the evaluation of PERR for traditional villages, the cultural protection is the basic premise. The evaluation is a key part of the PERR process, through which the utilization potential of traditional villages can be explored and confirmed. The evaluation conclusions are the basis for formulating the post-disaster reconstruction management plan and determining the reconstruction mode. When researching the indicator system for selecting the PERR mode for traditional villages, it was found that the importance of each indicator was difficult to determine, while the interconnection and interdependence of each indicator were relatively prominent. In order to be able to reflect the interrelatedness and interdependence among the indicators in the evaluation process, the DAP was constructed to determine the weights by the degree of dependence. The author applies the method to the following actual cases to inject more technical methods and rational thinking into the PERR of traditional villages and to test the scientific effectiveness and rationality of the method in this comprehensive evaluation of multi-level indicators.

## 4. Evaluation for selecting PERR modes of Xieluo-buzi traditional villages

### 4.1. Xieluo-Buzi Village, a traditional village in the M6.8 Luding earthquake affected area

**Figure 3:**
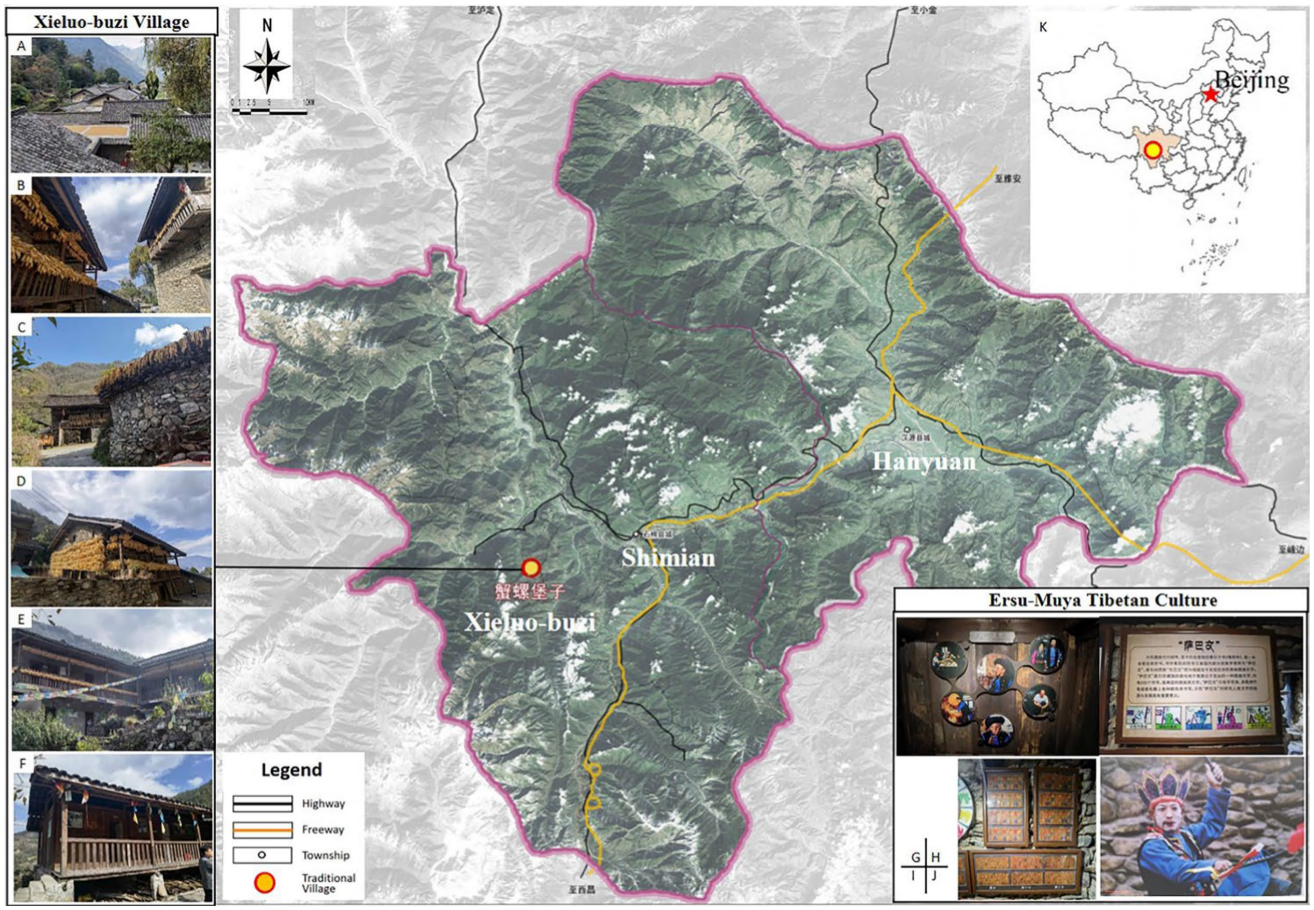
Location Map of Xieluo-buzi Village (Adapted from relevant information). **(A–F)** China traditional village: Xieluo-buzi Village (Photographed by the author); **(G–J)** Ersu-Muya Tibetan culture (The author filmed in the exhibition column of Diaolou); **(G)** Inheritor of the Ersu Tibetan; **(H)** The day counting book “Saba Wen” passed down from generation to generation by the Ersu Tibetan people; **(I)** Ersu Tibetan Calendar; **(J)** Ersu Tibetan traditional activities; **(K)** Location map (39).

Xieluo-buzi Village, the case study of this research, was included in the first batch of the Chinese traditional village list in 2013. It is located in Group 5, Jiangba Village, Xieluo Tibetan Township, Shimian County, Sichuan Province. It is a gathering place for Ersu Tibetan. The Ersu-Muya Tibetan culture is on the provincial-level non-material cultural heritage protection list (Figure 3). The village is built against the mountains, with rich landscape layers and distinctive stylistic features created during a historical period. Currently, the village comprises 78 households with 254 residents, and the population of the Ersu Tibetan ethnic group is 206. Xieluo-buzi possesses a unique indigenous Tibetan culture with its own pictographic writing, language, and traditions. The local production, lifestyle, and religious practices have always been passed down through generations. Thus far, this culture has been relatively well-preserved.

### 4.2. Disaster situation of Xieluo-Buzi village

**Figure 4:**
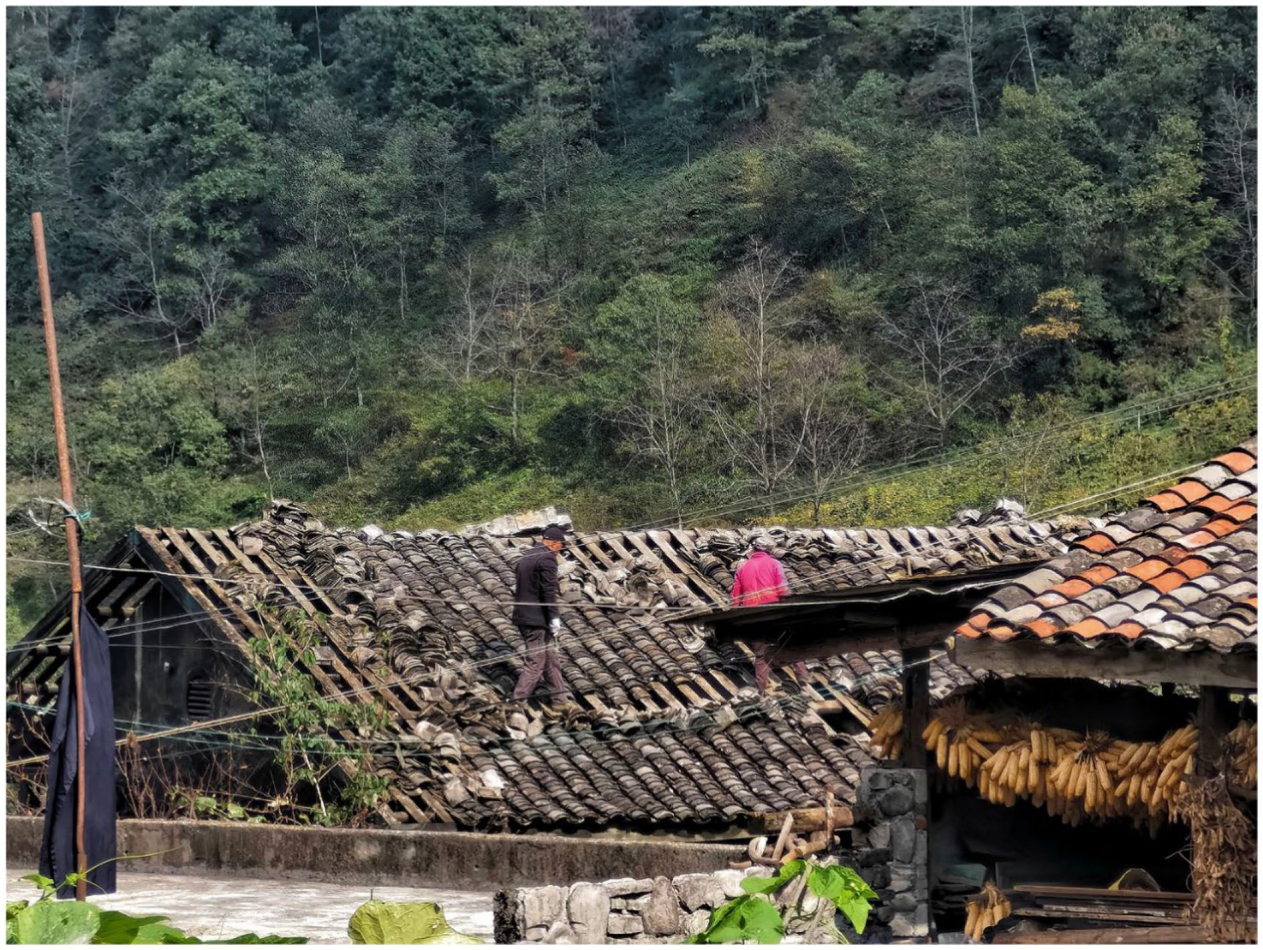
Traditional houses damaged by the “9.5” Luding Earthquake in Xiluo-buzi village (Photographed by the author).

**Figure 5:**
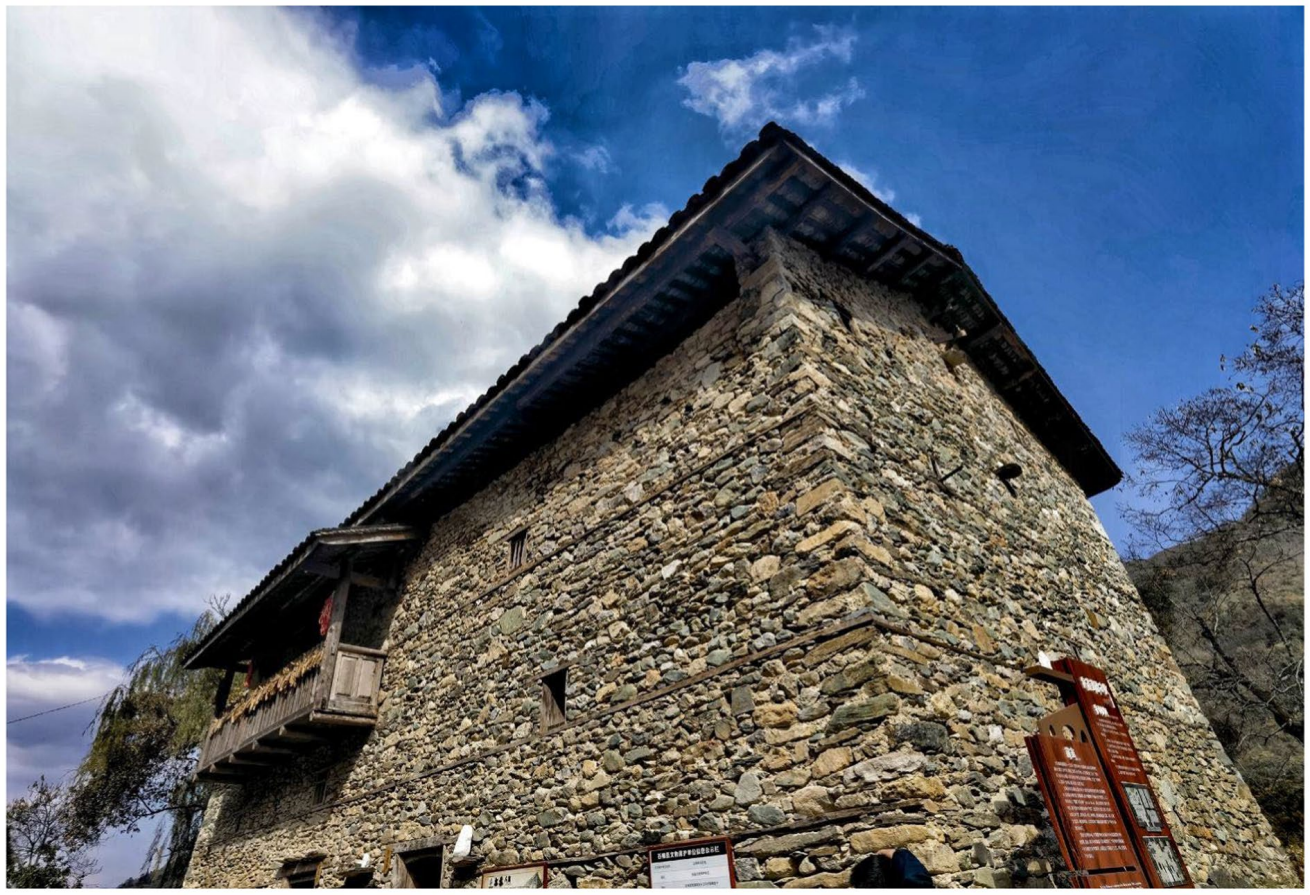
Diaolou in Xieluo-buzi village (Photographed by the author).

**Figure 6:**
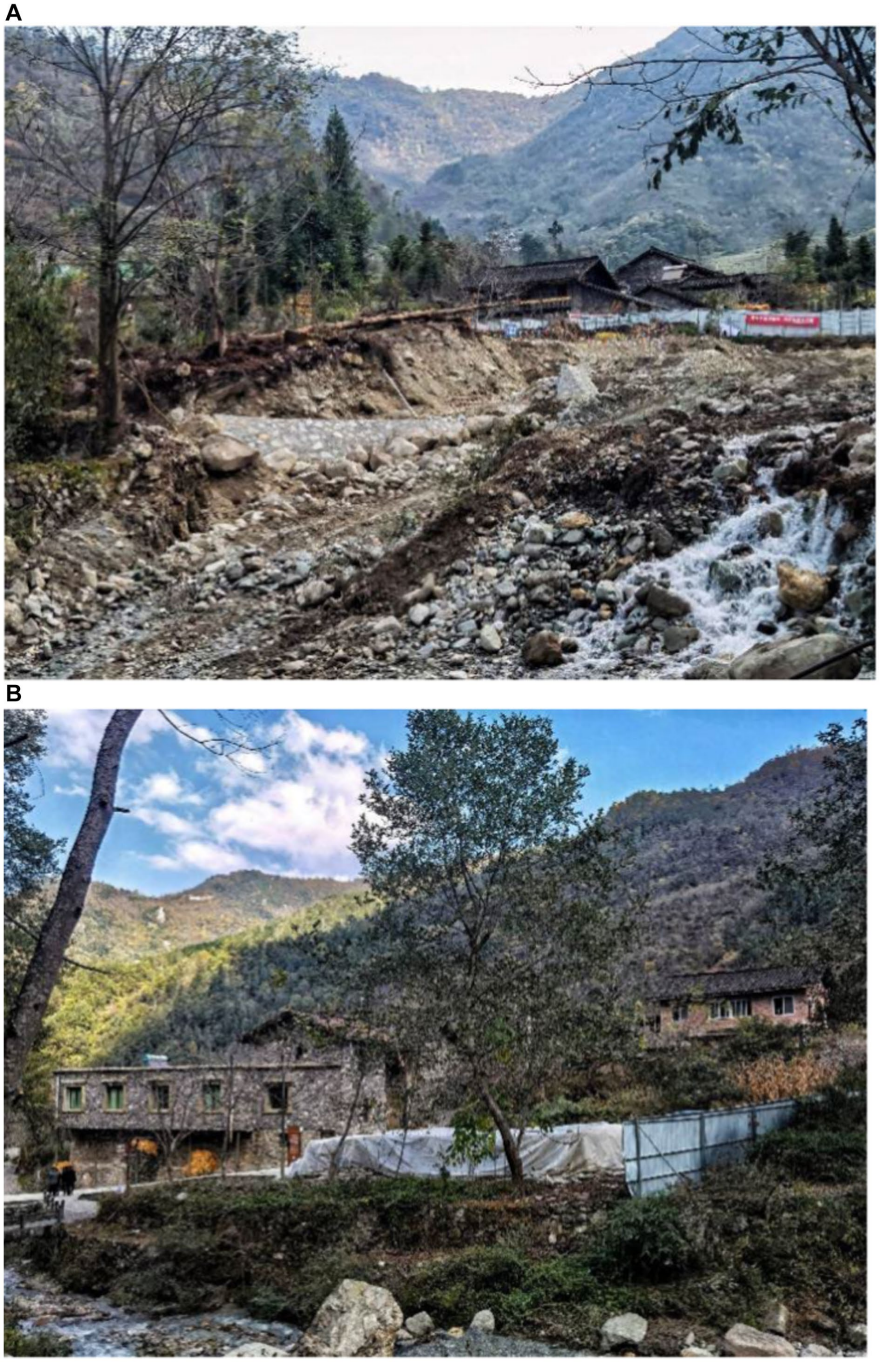
Post-disaster situation by the “9.5” Luding Earthquake in Xieluo-buzi village (Source. Photographed by the author): **(A)** Physical environment damaged by the “9.5” Luding Earthquake in Xieluo-buzi village; **(B)** Road and environment damaged by the “9.5” Luding Earthquake in Xieluo-buzi village.

On September 5, 2022, an Ms6.8 earthquake struck Luding Sichuan in western China, which particularly affected the county of Luding, Garze Tibetan Autonomous Prefecture, Sichuan Province, China. A quantity of cultural relic protection units and traditional village buildings were damaged and cracked, and the historical ecological environment was also destroyed. The *M*6.8 Luding earthquake caused partial damage to the traditional buildings of Xieluo-Buzi Village, with some bricks and tiles falling off (Figure 4). Although no widespread collapses can be observed, reinforcement is generally needed. Fortunately, the exterior of a former cultural heritage, which is protected by Cultural Relics Protection Units in Sichuan Province, and is a typical example of the Ersu-style architecture with a history of 300 years, remains undamaged (Figure 5). Other infrastructure and the physical environment in the village suffered varying degrees of damage (Figure 6). Overall, no remarkable change has occurred in the original site of Xieluo-buzi Village.

### 4.3. Evaluation for selecting PERR modes of Xieluo-buzi traditional villages

#### 4.3.1. Assignment of values to each level of the indicator system

In order to make a scientific evaluation of the support degree of the three reconstruction modes, the author followed the team to conduct a special field survey to understand the current situation of the disaster, the value of material and intangible cultural heritage of the villages, the natural geography and environmental conditions, randomly learned about the will of the villagers during the research, discussed with the relevant management departments and check the post-disaster reconstruction policies and safeguard related documents (40–42), and combined with the analysis of the three PERR modes in part II of this paper, to make a support-level evaluation. The higher the conservation level of the village (A1) is, the greater the support is for on-site restoration. If the traditional buildings and the integrity of the historical environment after the disaster (B1) are severely damaged and the authenticity of the village itself (B2) is lost, the support for off-site relocation is increased. The support level of three PERR modes of Xieluo-buzi Village is obtained (Table 5).

**Table 5:** The support level of three PERR models of Xieluo-buzi village.

Target layer	First-level	Second-level indicators	Support level
Mode Selection of post-disaster recovery and reconstruction of traditional village (G)	The Value of Traditional village cultural heritage (A)	Protection level of traditional villages	10/0/2
		Relevance to significant historical events, revolutionary movements, and famous figures	2/0/0
		Importance of ethnic culture	10/8/2
		The uniqueness of ethnic and local characteristics	8/4/2
		Representativeness of traditional village’s regional architectural style and spatial layout	8/0/4
		Uniqueness of local materials and craftsmanship	8/2/4
		Presence of inheritor of ethnic, cultural heritage	10/10/10
	Post-disaster situation of traditional village (B)	Integrity of traditional buildings and historical environment post-disaster	6/2/6
		Authenticity of the village	8/2/6
		Scale of the village	8/4/8
		Integrity of public facilities post-disaster	6/4/6
	Social capital of traditional village (C)	Mutual assistance and altruistic behavior within the village’s interpersonal relationship	8/8/4
		Influence and appeal of grassroots village organizations	8/8/4
		Influence of village elites	8/8/6
		Trust and emotional stability of villagers	6/8/6
		Execution capability of non-governmental organizations (NGOs)	0/0/0
		Support for mobilizing private capital	2/2/2
	Management and policy support in PERR (D)	Social impact of the disaster	4/4/4
		Policy support and guarantee for post-disaster reconstruction and rural revitalization	8/8/6
		Fairness of government policies	6/8/6
		Post-disaster execution of government departments	8/8/8
		Level of support from government reconstruction funds	4/6/6
		Trust of affected people in the government	8/8/8
	Physical geography and environmental condition (E)	Permissibility of geological hazard environment	6/8/8
		Accessibility of transportation	6/8/6
		Availability of land resources for reconstruction needs	4/6/4
		Ecological value of the village environment	6/6/6
	Development prospects and benefits (F)	Significant improvement in villagers’ living standards	6/8/6
		Significant increase in villagers’ happiness index	6/6/4
		Influence on the surrounding areas	6/6/4
		Reflection of heritage conservation value	10/2/6
		Value of village cultural tourism and future development planning potential	10/4/6

#### 4.3.2. Evaluation calculation and results

For the evaluation of the mode 1 of PERR for Xieluo-buzi Village, the dependence degree among indicators determines their importance, that is, a higher support level indicates a greater influence of the indicator. Based on the dependency analysis for the secondary indicators within the first-level indicator Value of Traditional Village Cultural Heritage (A), the following correlation table is obtained (Table 6).

**Table 6:** The interdependency among the indicators in the value of traditional village cultural heritage (A).

	A1	A2	A3	A4	A5	A6	A7
A1	1	0.1	0.1	0.1	0.1	0.1	0.1
A2	0.1	1	0	0	0	0	0
A3	0.1	0	1	0.3	0	0	0.2
A4	0.1	0	0.1	1	0.1	0.3	0.1
A5	0.1	0	0	0.1	1	0.3	0
A6	0.1	0	0	0.1	0.1	1	0
A7	0.1	0	0.2	0	0	0	1

In the first-level indicator of the value of traditional villages’ cultural heritage (A), the dependence of the second-level indicator of the uniqueness of ethnic and local characteristics (A4) on the importance of ethnic culture (A3) is 0.1; the dependence of A3 on A4 is 0.3, and the correlation degree between A3 and A4 is 0.2. Therefore, the dependency matrix for the second-level indicators within the first-level indicator A is calculated as follows:


(8) $$A=\left(\begin{array}{ccccccc} 1 & 0.1 & 0.1 & 0.1 & 0.1 & 0.1 & 0.1\\ 0.1 & 1 & 0 & 0 & 0 & 0 & 0\\ 0.1 & 0 & 1 & 0.2 & 0 & 0 & 0.2\\ 0.1 & 0 & 0.2 & 1 & 0,1 & 0.2 & 0.05\\ 0.1 & 0 & 0 & 0.1 & 1 & 0.2 & 0\\ 0.1 & 0 & 0 & 0.2 & 0.2 & 1 & 0\\ 0.1 & 0 & 0.2 & 0.05 & 0 & 0 & 1 \end{array}\right)$$

Calculate the sum of interdependence between an indicator and other indicators, and sort them in descending order:


(9) $$\begin{array}{l} c_{1}=b_{4}=0.65,c_{2}=b_{1}=0.6,c_{3}=b_{3}=0.5,c_{4}=b_{6}=0.5,\\ c_{5}=b_{5}=0.5,c_{6}=b_{7}=0.35,c_{7}=b_{2}=0.1 \end{array}$$

Therefore, the indexes are ranked as $a_{4},a_{1},a_{3},a_{6},a_{5},a_{7},a_{2}$ based on the degree of interdependence.

The eigenvalues of matrix A in descending order are:


(10) $$\begin{array}{l} \lambda_{1}=1.5023,\lambda_{2}=1.2009,\lambda_{3}=1.0554,\lambda_{4}=0.9177,\\ \lambda_{5}=0.8291,\lambda_{6}=0.8000,\lambda_{7}=0.6946 \end{array}$$

The weight vector for the second-level indicators is:


(11) β1=λ1∑i=17λ1=0.2145,β2=0.1716,β3=0.1508,β4=0.1311,β5=0.1184,β6=0.1143,β7=0.0992

The support for the first-level indicator “Value of Traditional Village Cultural Heritage (A)” is:


(12) $$\begin{array}{l} Y_{1}=\beta_{1}a_{4}+\beta_{2}a_{1}+\beta_{3}a_{3}+\beta_{4}a_{6}+\beta_{5}a_{5}+\beta_{6}a_{7}\\ +\beta_{7}a_{2}=0.2145\times 8+0.1716\times 10+0.1508\\ \times 10+0.1311\times 8+0.1184\times 8+0.1143\times 10\\ +0.0992\times 2=8.2774 \end{array}$$

Using the same way, the correlation dependency between the second-level indicators among the first-level indicators is (Table 7).

**Table 7:** The interdependency among the second-level indicators.

The interdependency among the indicators in post-disaster situation of traditional village (B)				
	B1	B2	B3	B4
B1	1	0.2	0.3	0.2
B2	0.3	1	0	0.1
B3	0.1	0	1	0
B4	0.2	0	0.1	1

The interdependency among the indicators in social capital of traditional village (C)						
	C1	C2	C3	C4	C5	C6
C1	1	0	0,1	0.2	0	0.1
C2	0.3	1	0.1	0.1	0.1	0.1
C3	0.2	0.1	1	0.1	0	0.2
C4	0.2	0.1	0.3	1	0	0.1
C5	0	0	0.1	0.1	1	0.2
C6	0.2	0.1	0.3	0.1	0,1	1

The interdependency among the indicators in management and policy support in PERR (D)						
	D1	D2	D3	D4	D5	D6
D1	1	0	0	0.2	0	0
D2	0	1	0.1	0.1	0.1	0
D3	0	0.1	1	0.1	0.1	0
D4	0.2	0.2	0.2	1	0.2	0.1
D5	0.1	0.2	0.1	0.1	1	0
D6	0.1	0.1	0.3	0.3	0.1	1

The interdependency among the indicators in physical geography and environmental condition (E)				
	E1	E2	E3	E4
E1	1	0	0	0
E2	0.1	1	0	0.1
E3	0.3	0.2	1	0
E4	0.1	0	0.2	1

The interdependency among the indicators in development prospects and benefits (F)					
	F1	F2	F3	F4	F5
F1	1	0	0.1	0	0.2
F2	0.3	1	0.2	0.1	0.2
F3	0.1	0.1	1	0.2	0.2
F4	0	0	0.1	1	0.3
F5	0.1	0	0.1	0.3	1

The dependency matrices determined by the second-level indicators in other first-level indicators are:


(13) $$B=\left(\begin{array}{cccc} 1 & 0.25 & 0.2 & 0.2\\ 0.25 & 1 & 0 & 0.05\\ 0.2 & 0 & 1 & 0.05\\ 0.2 & 0.05 & 0.05 & 1 \end{array}\right)$$

(14) $$C=\left(\begin{array}{cccccc} 1 & 0.2 & 0.15 & 0.2 & 0 & 0.15\\ 0.2 & 1 & 0.1 & 0.1 & 0.05 & 0.1\\ 0.15 & 0.1 & 1 & 0.2 & 0.05 & 0.25\\ 0.2 & 0.1 & 0.2 & 1 & 0.05 & 0.1\\ 0 & 0.05 & 0.05 & 0.05 & 1 & 0.15\\ 0.15 & 0.1 & 0.25 & 0.1 & 0.15 & 1 \end{array}\right)$$

(15) $$D=\left(\begin{array}{cccccc} 1 & 0 & 0 & 0.2 & 0.05 & 0.05\\ 0 & 1 & 0.1 & 0.15 & 0.1 & 0.05\\ 0 & 0.1 & 1 & 0.15 & 0.1 & 0.15\\ 0.2 & 0.15 & 0.15 & 1 & 0.15 & 0.2\\ 0.05 & 0.1 & 0.1 & 0.15 & 1 & 0.05\\ 0.05 & 0.05 & 0.15 & 0.2 & 0.05 & 1 \end{array}\right)$$

(16) $$E=\left(\begin{array}{cccc} 1 & 0.05 & 0.2 & 0.05\\ 0.05 & 1 & 0.1 & 0.05\\ 0.2 & 0.1 & 1 & 0.1\\ 0.05 & 0.05 & 0.1 & 1 \end{array}\right)$$

(17) $$F=\left(\begin{array}{ccccc} 1 & 0.2 & 0.1 & 0 & 0.15\\ 0.2 & 1 & 0.15 & 0.05 & 0.1\\ 0.1 & 0.15 & 1 & 0.15 & 0.15\\ 0 & 0.05 & 0.15 & 1 & 0.3\\ 0.15 & 0.1 & 0.15 & 0.3 & 1 \end{array}\right)$$

calculate that:


(18) $$Y_{2}=6.421,Y_{3}=5.7332,Y_{4}=6.6654,Y_{5}=5.353,Y_{6}=7.876$$

Material factors, such as the heritage value, degree of damage, and socioeconomic benefits of the traditional village, influence the policy direction. The formulation of policies also needs to consider the residents’ actual demands comprehensively. The trust and support of public relations positively affect the restoration of socioeconomic order in disaster-stricken areas. In addition, the strong willingness of the people, which is a controlled variable, ultimately determines the PERR mode selection. The dependency matrix of the first-level indicator is determined by analyzing the relationships and influences among these indicators (Table 8).

**Table 8:** The interdependency among the first-level indicators.

	A	B	C	D	E	F
A	1	0	0.4	0.1	0.1	0.2
B	0	1	0	0	0	0
C	0.2	0	1	0.3	0.1	0.2
D	0.2	0.2	0.3	1	0.1	0.1
E	0	0.1	0	0.1	1	0
F	0.2	0.1	0.2	0.2	0.2	1

The dependency matrix for the first-level indicators is calculated as follows:


(19) $$G=\left(\begin{array}{cccccc} 1 & 0 & 0.3 & 0.15 & 0.05 & 0.2\\ 0 & 1 & 0 & 0.1 & 0.05 & 0.05\\ 0.3 & 0 & 1 & 0.3 & 0.05 & 0.2\\ 0.15 & 0.1 & 0.3 & 1 & 0.1 & 0.15\\ 0.05 & 0.05 & 0.05 & 0.1 & 1 & 0.1\\ 0.2 & 0.05 & 0.2 & 0.15 & 0.1 & 1 \end{array}\right)$$

using the same method as above for the target layer, the total support level for selecting mode 1 is calculated: Y=6.4105 .

Similarly, the support levels for three modes are calculated (Table 9).

**Table 9:** Support level of three modes of PERR of Xieluo-buzi village.

Title 1	Mode 1	Mode 2	Mode 3
The value of traditional village cultural heritage	$Y_{1}=8.2774$	$Y_{1}=3.4696$	$Y_{1}=3.2148$
Post-disaster situation of traditional village	$Y_{2}=6.421$	$Y_{2}=2.659$	$Y_{2}=5.9206$
Social capital of traditional village	$Y_{3}=5.7332$	$Y_{3}=6.0244$	$Y_{3}=4.031$
Management and policy support in PERR	$Y_{4}=6.6654$	$Y_{4}=7.3158$	$Y_{4}=6.6884$
Physical geography and environmental condition	$Y_{5}=5.353$	$Y_{5}=6.959$	$Y_{5}=5.837$
Development prospects and benefits	$Y_{6}=7.876$	$Y_{6}=5.0088$	$Y_{6}=5.2026$
Target layer support	Y=6.4105	Y=5.3958	Y=4.9611

According to the above calculation results, mode 1 has the highest support, followed by mode 2, then mode 3. Therefore, it is recommended to carry out mode 1 for Xieluo-buzi Village, which protect the material and intangible cultural heritage of the national traditional village to a greater extent, to preserve the original village form, and to avoid the deterioration of the original site due to relocation, and the change of the traditional spatial pattern due to organic evacuation, which will lead to the reduction or extinction of its comprehensive heritage value. However, the final decision still depends on ε , which is the comprehensive determination of possible control variables.

## 5. Discussion

### 5.1. Innovations

Compared with other studies, the contribution of this paper lies in the following four aspects. Firstly, according to the research results of other scholars, three modes of PERR of traditional villages are summarized and planned. Second, it deeply analyzes the influencing factors of PERR mode selection for traditional villages based on heritage value, and an indicator system for PERR mode selection for traditional villages was constructed. Thirdly, the author summarizes other commonly used evaluation methods and creates the DAP, which is a new method to determine the weight vectors. The scientific value and significance of this method is that it overcomes the drawbacks of many evaluation methods that need to determine the weight according to the importance of the indicators, which is difficult to determine in many cases. Fourthly, according to the evaluation results, it is targeted to provide decision-making suggestions for the PERR of traditional villages. The research in this paper hopes to provide scientific guidance and a useful reference for disaster prevention and mitigation and deliver recovery efforts in earthquake-stricken areas. At the same time, it is hoped that the further application of the DAP, its superiority and practicality will be further reflected. The starting point and level of the study are profoundly innovative.

### 5.2. PERR of traditional villages

From this study, it can be seen that: (1) for traditional villages with a high level of protection and no destructive damage to the original site, on-site restoration is the most important mode of choice, which retains the most spatial characteristics of traditional ethnic settlements, (2) the village’s own network of the social capital relationship and policy orientation also determines the direction of choice for PERR of villages, (3) whether the geographic environment in which villages are situated avoids active faults is usually regarded as an important basis for the selection of sites for PERR to avoid recurrence of disaster risks, which will lead to further damage to the lives and properties of indigenous people and village ethnic heritage, (4) on the basis of respecting the indigenous people’s ethnic beliefs and traditional living spaces, their aspirations for a better life cannot be ignored, and remodeling and promoting ethnic cultural tourism and enhancing the satisfaction and happiness of their lives are the direction of development that the indigenous people look forward to after PERR.

Based on the above points, the authors believe that in the PERR of traditional villages, first of all, in terms of research, it is necessary to conduct in-depth investigations to understand the value of the material and intangible cultural heritage, the characteristics of the regional and ethnic cultures, the historical information and the ecological resources of the traditional villages, and make a real record of the current situation after the disaster, which is implemented to each household and each aboriginal and each village residence, and determine the technical measures for the restoration of the current situation, and understand the behavioral and psychological needs and expectations of indigenous people. Moreover, it analyzes the impacts of the PERR on the production and living space and the ethnic culture of the village, and understand the opportunities and challenges these impacts will bring to the future development direction of the villages. Secondly, respect the principles of authenticity and integrity of heritage conservation. The traditional residence, production and living space and the natural ecological environment on which the village depends should be protected in a holistic way. The authenticity of the original village residence should be rebuilt with local raw materials as much as possible, and the spatial pattern of the village should be protected in an original way. Finally, promote the activation and utilization of village heritage, form their own advantages of ethnic industries, and create a distinctive ethnic cultural industry system relying on ethnic, cultural characteristics so as to promote the economic and social after PERR. It can be seen that the protection of traditional villages is a dynamic protection process. In this process, it is necessary to respect the inclinations of the indigenous people, in which concerned about optimizing residential functions to meet the needs of modern lives. However, when their residences become heritage sites to be protected, their aspiration for a better life may be ignored, and they may lose the dominant power over their own houses. Therefore, in PERR, it is clear that indigenous people are the main body of construction and management, and the interests of all parties should be harmonized, the social network of the indigenous people is maintained, and the fair rights of the indigenous people to participate in the implementation of the project and to enjoy the preferential policies and resource benefits are guaranteed, as they are the real main body of the heritage protection so that the traditional village ethnic heritage can be inherited and developed permanently in the protection.

### 5.3. Insufficient understanding of traditional village heritage value

The research indicates that the indigenous people have an insufficient understanding of the value of traditional village heritage, leading to a lack of emphasis on the restoration and improvement of their own traditional houses. This lack of understanding and the absence of public participation, which are regarded as control variables in the model, may alter the selection of PERR modes. This scenario leads to the loss of the traditional character of a village and ultimately turns historical heritage into historical regret. Therefore, popularizing disaster preparedness education, raising awareness of the value and conservation of cultural heritage, and strengthening the seismic resistance of traditional houses in PERR are crucial.

### 5.4. Validity of the method

The authors propose a new method, namely Dependency Analytic Process (DAP), which differs from other evaluation methods. This method has good adaptability when interrelations and interdependency exist among evaluation indicators. Compared with AHP, DAP has the following advantages: (1) in AHP, the judgment matrix is based on the indicators’ importance, which is determined by subjective factors. By contrast, in DAP, the construction of the dependency matrix is based on the interdependence between indicators, which is determined by the connotation of the indicators and is relatively objective, (2) AHP requires consistency in the judgment matrix, and the consistency test is relatively cumbersome. On the contrary, the dependency matrix of DAP is a diagonally dominant real symmetric matrix. Therefore, it is a positive-definite matrix, and its eigenvalues are all positive real numbers, simplifying the discrimination method, (3) in AHP, the judgment matrix determines the weight based on the eigenvectors of the eigenvalues. However, in the dependency matrix of DAP, the weight is determined based on the eigenvalues. From the perspective of the matrix as a linear transformation, the magnitude of eigenvalues is the scaling of vectors in the feature subspace. The more important an indicator is, the larger its weight should be. Moreover, the explicitness of the causal logical relationship is increased. The DAP introduces the correlation and interdependence among the indicators into the evaluation process, which is applicable to the comprehensive evaluation of multiple factors, and it is especially in situations when the importance of indicators is difficult to distinguish. In this paper, due to the interdependence and importance of each element, the results obtained are more in line with reality. The scientific value and significance of this method is that it overcomes the drawbacks of many evaluation methods that require the determination of weights according to the importance of indicators. It is a new method for determining the weight vectors, which can be combined with other comprehensive evaluation methods which involve weight vectors in the evaluation process and has a broad application prospect.

The validity of the method needs to be further verified. The authors have explored the application of the DAP in the selection of PERR modes for traditional villages and verified the effectiveness of the method. Since a large amount of actual research work is required to conduct an assessment, it is believed that with the widespread applications of the DAP, its superiority and practicality will be further demonstrated.

### 5.5. Research limitations

The indicator system determined in this study was constructed by the author based on research results and judgments through extensive reading of relevant literature and field research, especially the assignment of more subjective evaluation components. Therefore, there is still room for optimization and refinement of the various components of the system, such as the determination of evaluation indicators, the application of evaluation methods, and the formulation of evaluation standards, etc., which require more authoritative experts’ decision-making support and more empirical evidence of project evaluation to be more scientifically corrected and improved.

## 6. Conclusion

This paper established an evaluation and analysis method named DAP, and initially constructed a relatively complete indicator system for the comprehensive evaluation of traditional village PERR mode selection. This indicator system was used for empirical analysis by taking the Xieluo-buzi Village, which is rich in material and intangible cultural heritage in the *M*6.8 Luding earthquake-stricken area, as an example. The author field research to investigate the current situation of the disaster, the value of village heritage, the potential for development and utilization, etc., through the DAP to get the optimal choice of on-site restoration. The historical ecological environment and spatial pattern of Xieluo-buzi have not been seriously damaged after the earthquake, and the base is not in the fracture zone, and the transportation and other infrastructures have a certain foundation, so the scientific on-site restoration can protect the cultural heritage of the village to the greatest extent.

The results are expected to provide a basis for selecting the PERR mode for traditional villages in China and decision-making for PERR. It is also expected to provide a valuable reference for the post-disaster reconstruction planning in the historical area affected by the recent major earthquake in Turkey.

## Data availability statement

The original contributions presented in the study are included in the article/supplementary material, further inquiries can be directed to the corresponding author/s.

## Author contributions

CH: conceptualization, formal analysis, writing—original draft preparation, and visualization. CH and TH: methodology and data curation. TH: software. CH, JQ, and TH: validation. CH and JQ: investigation, resources, and writing—review and editing. JQ: supervision, project administration and funding acquisition. All authors have read and agreed to the published version of the manuscript. All authors contributed to the article and approved the submitted version.

## Funding

This research was funded by the National Natural Science Foundation of China under the project “Research on Urban Spatial Coupling Mechanism Between Urban Epidemic Spreading and Vulnerability and Planning Response in Chengdu-Chongqing Area” (Grant No. 52078423); The Major Program of Sichuan Provincial Scientific Research under the Project of “Research and Demonstration of Resilient Collaborative Planning and Design for Park Cities” (Grant No. 2020YFS0054); Sichuan Provincial Science and Technology Innovation Platform and Talent Plan “Research on the Construction and Development Strategies of Several Major Infrastructure Systems for New Smart Cities” (Grant No. 2022JDR0356).

## Conflict of interest

The authors declare that the research was conducted in the absence of any commercial or financial relationships that could be construed as a potential conflict of interest.

## Publisher’s note

All claims expressed in this article are solely those of the authors and do not necessarily represent those of their affiliated organizations, or those of the publisher, the editors and the reviewers. Any product that may be evaluated in this article, or claim that may be made by its manufacturer, is not guaranteed or endorsed by the publisher.
